# Dr. Harrison Allen

**Published:** 1897-12

**Authors:** 


### DR. HARRISON ALLEN.

Dr. Harrison Allen, of Philadelphia, died November 14, 1897, of angina pectoris.

Dr. Allen was born in 1841, and completed his preliminary educational training in the Central High School, graduating in 1856, and subsequently entered the Department of Medicine, University of Pennsylvania, graduating from that institution in 1861. From 1862 to 1865 he was assistant surgeon in the army, being stationed principally at Washington, D. C. He held the rank of brevet-major in 1865.

When Dr. George A. Wood established the Auxiliary Department of Medicine in the University of Pennsylvania, Dr. Allen, then twenty-four years old, was called to the chair of Comparative

Anatomy and Zoology; his previous work in the Smithsonian Institution having impressed Dr. Wood with his ability. From 1866 to 1878 he was professor of Anatomy in the Philadelphia Dental College. In the year 1878 he was offered and accepted the chair of Physiology in the Department of Medicine, University of Pennsylvania. He resigned this in 1883. He was visiting surgeon of the Philadelphia Hospital from 1874 to 1878, assistant surgeon at Wills Eye Hospital from 1868 to 1870, and at St. Joseph's from 1870 to 1878. In 1894 he was made director of the Wistar Institute of Anatomy, founded by Dr. Isaac J. Wistar, and continued in this position until 1894. Dr. Allen enjoyed a large practice as specialist in diseases of the throat and lungs.

While he thus occupied positions of great responsibility, it was through his original research that he acquired an assured position as an authority in scientific circles. A warm personal friend of Professors Leidy and Cope, he possessed, in common with these distinguished men, an indefatigable determination to reach absolute truth in his particular domain of investigation.

His writings cover many subjects, having written about a hundred monographs, papers, and books on many phases of medicine. Among them are "A Monograph on the North American Bats;" "Outlines of Lectures on Comparative Anatomy;" "Studies in Facial Region;" "On the Life Form in Art;" "System of Human Anatomy;" "The Stage of the Development of the Bat," and "Clinical Study of the Skull." His works on the diseases of the nose and throat, of which he is one of the most eminent of specialists, are very numerous.

He was a member of many learned societies in and outside of medicine.

Dr. Allen's death at a comparatively early age has cast a shadow over scientific circles in Philadelphia, for the losses there have been very heavy in the past two or three years.

His funeral took place November 17, from his residence, and was attended by a large concourse. His body was incinerated at the Germantown Crematory in accordance with Dr. Allen's previously expressed desire.

